# Mass Sensitivity Optimization of a Surface Acoustic Wave Sensor Incorporating a Resonator Configuration

**DOI:** 10.3390/s16040562

**Published:** 2016-04-20

**Authors:** Wenchang Hao, Jiuling Liu, Minghua Liu, Yong Liang, Shitang He

**Affiliations:** Institute of Acoustics, Chinese Academy of Sciences, No.21, North 4th Ring West Road, Beijing 100190, China; haowenchang12@mails.ucas.ac.cn (W.H.); liuminghua@mail.ioa.ac.cn (M.L.); liangyong@mail.ioa.ac.cn (Y.L.); heshitang@mail.ioa.ac.cn (S.H.)

**Keywords:** mass sensitivity, Rayleigh surface acoustic wave (R-SAW) resonator, finite element method (FEM), coupling-of-modes (COM), sensitive areas

## Abstract

The effect of the sensitive area of the two-port resonator configuration on the mass sensitivity of a Rayleigh surface acoustic wave (R-SAW) sensor was investigated theoretically, and verified in experiments. A theoretical model utilizing a 3-dimensional finite element method (FEM) approach was established to extract the coupling-of-modes (COM) parameters in the absence and presence of mass loading covering the electrode structures. The COM model was used to simulate the frequency response of an R-SAW resonator by a *P*-matrix cascading technique. Cascading the *P*-matrixes of unloaded areas with mass loaded areas, the sensitivity for different sensitive areas was obtained by analyzing the frequency shift. The performance of the sensitivity analysis was confirmed by the measured responses from the silicon dioxide (SiO_2_) deposited on different sensitive areas of R-SAW resonators. It is shown that the mass sensitivity varies strongly for different sensitive areas, and the optimal sensitive area lies towards the center of the device.

## 1. Introduction

Since first reported by Venema in 1986 [1], the surface acoustic wave (SAW) resonator configuration for mass sensor applications has long been studied. The mass sensor was operated by applying a perturbation on SAW propagation using the mass loading effect [2]. Compared with the delay-line configuration used in SAW sensors, the resonator attracts more interest because it offers low loss and high Q-value, which benefits the improvement of the detection limit in mass sensing [3,4]. The mass sensitivity was investigated by analyzing the acoustic wave propagation in a layered structure consisting of a homogeneous thin film over a semi-infinite piezoelectric substrate [5,6]. Obviously, the design of the resonator plays a significant role in the improvement of the mass sensitivity, especially the sensitive area, which provides a lower detection limit when optimal design was performed in SAW gas chromatogram (SAW-GC) sensors [7], and larger sensitivity and cost reductions for SAW chemical sensors. However, it is a gap in the studies of the mass sensitivity associated with the sensitive areas of the SAW devices.

In 2001, Harding *et al.* presented a detailed experimental study of the mass sensitivity for Love-wave delay-line devices [8], which indicated the relative mass sensitivity was located at the IDT electrodes and the ‘sweet spot’ (between the IDTs) strongly depended on the guiding layer thickness, but, only qualitative results were obtained. Afterwards, further research on the spatial sensitivity distribution for Love-wave resonator devices was pursued by Powell *et al.* [9]. A rigorous boundary element method (BEM) and coupling-of-modes (COM) model was utilized to study the layered structure with embedded electrodes, quantifying the spatial sensitivity distribution effect. Their results demonstrated that the mass sensitivity of location variation is much greater than described in Harding’s previous report. Although Powell’s approach provides a good way to analyse the spatial sensitivity distribution, a very complicated derivation was needed to extract the COM parameters from BEM and the matrix eigenoperator. Owing to the fact it provides an easy way of analyzing complicated geometries, the finite element method (FEM) has proved to be a good tool to derive the influence of geometrical variations of the electrode shape and the surface perturbation of the sensors [10,11]. Therefore, the FEM with the commercial software COMSOL MULTIPHYSICS was utilized in our work to model the structures in the absence and presence of mass loading covering the electrodes to extract the COM parameters.

**Figure 1:**
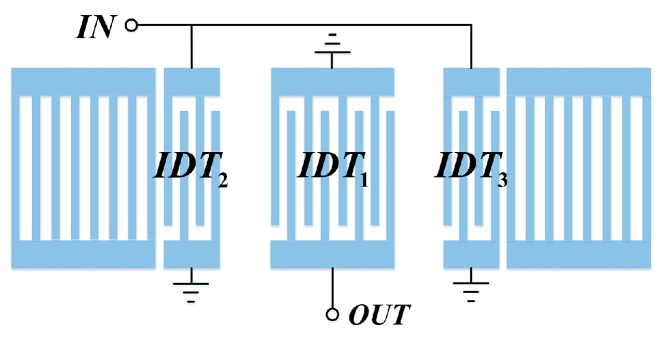
The schematic of a two-port R-SAW resonator with three IDTs.

In this contribution, a two-port Rayleigh SAW (R-SAW) resonator structure with three IDTs was used to analysis the mass sensitivity. The schematic of the resonator is depicted in Figure 1. The resonator cavity is determined by three IDTs and two identical shorted grating reflectors, in which the lateral IDTs are symmetrically arranged with respect to the center IDT [12]. Here, the resonator structure was assumed to be symmetric, and the common electrical port of IDT_2_ and IDT_3_ was designated as an input port and the electrical port of IDT_1_ as an output port. The SAW generated by the input port at a particular frequency is constructively reflected by the reflectors close to the same period of the IDTs, forming a resonator cavity. The two-port resonator will only excite the first and the third SAW longitudinal modes, realizing the characteristics of low insertion loss and narrow-band.

Then, the effect of the position of the sensitive areas in the resonator on mass sensitivity was analyzed by using the theoretical layered structure approach and verified by experiments. In the theoretical model, the COM parameters in the structures with or without mass loading covering the electrodes were extracted by 3-D FEM using COMSOL MULTIPHYSICS. Then the frequency responses of the resonator with mass loading at various sensitive areas were simulated by using the COM model. By computing the frequency shift of the resonator, the mass sensitivity variation dependence on the sensitive areas was studied, and hence an optimal design of the resonator for mass sensor applications was obtained. The corresponding experiments were implemented to validate the theoretical results.

## 2. Theoretical Analysis

### 2.1. COM Equations for SAW Resonator

**Figure 2:**
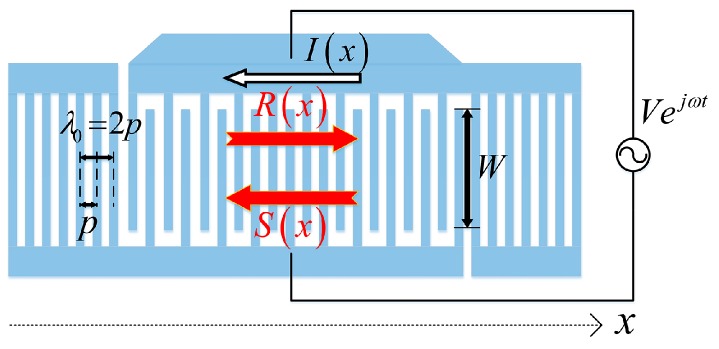
The schematic of the COM model for typical resonator configuration.

COM provides an effective approach for modeling and simulating SAW devices with different structures [13]. The schematic diagram of the COM model for a typical resonator configuration is shown in Figure 2.

An alternating driven voltage *V* connected to the bus bar excites acoustic waves. The waves are described by *R*(*x*) and *S*(*x*), representing modes propagating in the positive and negative *x*-directions, respectively. In reverse, the waves propagating under the electrodes cause a flow of the current *I*. *λ*_0_ is the electrical period, namely, the wavelength. For a uniform structure, *λ*_0_ equals to 2*p*, where *p* is the structural period and *W* is the acoustic aperture. By introducing the slowly varying scalar fields *R*_0_(*x*) and *S*_0_(*x*) as follows:

$$\left\{ \begin{array}{l} R\left(x\right)=R_{0}\left(x\right)\text{exp}\left(j\omega t-jkx\right)\\ S\left(x\right)=S_{0}\left(x\right)\text{exp}\left(j\omega t+jkx\right) \end{array}\right.$$

the COM equations of IDT assume the form:

(1) $$\left\{ \begin{array}{l} \frac{dR_{0}}{dx}=j\kappa S_{0}\text{exp}\left(2j\Delta x\right)+j\alpha V\text{exp}\left(j\Delta x\right)\\ \frac{dS_{0}}{dx}=-j\kappa^{*}R_{0}\text{exp}\left(-2j\Delta x\right)-j\alpha^{*}V\text{exp}\left(-j\Delta x\right)\\ \frac{dI}{dx}=-2j\alpha^{*}R_{0}\text{exp}\left(-j\Delta x\right)-2j\alpha S_{0}\text{exp}\left(j\Delta x\right)+j\omega CV \end{array}\right.$$

where ∆ is the detuning parameter:

$$\Delta =k-k_{0}=k_{r}-k_{0}-j\gamma =\frac{\omega }{v}-k_{0}-j\gamma$$

*k* is the complex wave number, *k* = *k*_r_ − j*γ*, and *k*_0_ is the synchronous wave number. The independent parameters of the model are velocity *v*, reflectivity *κ*, transduction coefficient *α*, static capacitance per unit length *C* and propagation attenuation *γ* (superscript * denotes the complex conjugate). Usually, *κ* and *α* are complex numbers. However, for bidirectional uniform structures, the *κ* and *α* are chosen real-valued. Additionally, when the R-SAW is considered, *γ* is ignored as lossless propagation.

Considering the case of periodic shorted-grating, the excitation source is omitted, namely *V* = 0 [14]. The homogeneous form of the COM Equation (1) is obtained:

(2) $$\left\{ \begin{array}{l} \frac{dR_{0}}{dx}=j\kappa S_{0}\text{exp}\left(2j\Delta x\right)\\ \frac{dS_{0}}{dx}=-j\kappa^{*}R_{0}\text{exp}\left(-2j\Delta x\right) \end{array}\right.$$

The general solutions of the Equation (2) are:

$$\left\{ \begin{array}{l} R_{0}=A_{+}\text{exp}\left[j\left(\Delta +D\right)x\right]+A_{-}\text{exp}\left[j\left(\Delta -D\right)x\right]\\ S_{0}=\frac{1}{\kappa }\left\{A_{+}\left(\Delta +D\right)\text{exp}\left[-j\left(\Delta -D\right)x\right]+A_{-}\left(\Delta -D\right)\text{exp}\left[-j\left(\Delta +D\right)x\right]\right\} \end{array}\right.$$

where $A_{+}$ and $A_{-}$ are undetermined coefficients, identified by the boundary conditions. Here, *D* is the dispersion relation for a eigenwave in shorted-grating:

$$D=\sqrt{\Delta^{2}-{\left|\kappa \right|}^{2}}$$

It is nondispersive for a surface acoustic wave propagating on both free and metallized surface of piezoelectric crystals. However, in periodic electrodes structures, the surface impedance becomes discontinuous as the alternation of free and metallized areas, causing the dispersive phenomenon. For the R-SAW case (Δ = (ω/*v*) – *k*_0_) the stopband appears in the frequency domain of $f_{0}\left(1-\frac{\left|\kappa \right|}{k_{0}}\right)<f<f_{0}\left(1+\frac{\left|\kappa \right|}{k_{0}}\right)$ with serious attenuation, where *D* is purely imaginary. The lower and upper edges of the stopband are found when *D* is zero as follows:

(3) $$\{\begin{array}{c} f_{sc-}=f_{0}\left(1-\frac{\left|\kappa \right|}{k_{0}}\right)\\ f_{sc+}=f_{0}\left(1+\frac{\left|\kappa \right|}{k_{0}}\right) \end{array}$$

Considering the case of periodic open-grating, the electrode current is zero, namely *I* = 0. The COM equations derived in the similar form as Equation (2) are:

$$\left\{ \begin{array}{l} \frac{dR_{0}}{dx}=j\left(\kappa +\frac{2\alpha^{2}}{\omega C}\right)S_{0}\text{exp}\left[2j\left(\Delta -\frac{2{\left|\alpha \right|}^{2}}{\omega C}\right)x\right]\\ \frac{dS_{0}}{dx}=-j\left(\kappa^{*}+\frac{2\alpha^{*2}}{\omega C}\right)R_{0}\text{exp}\left[-2j\left(\Delta -\frac{2{\left|\alpha \right|}^{2}}{\omega C}\right)x\right] \end{array}\right.$$

Introducing the equivalent detuning parameter ∆_oc_ and equivalent reflectivity *κ*_oc_:

$$\left\{ \begin{array}{l} \Delta_{oc}=\Delta -\frac{2{\left|\alpha \right|}^{2}}{\omega C}\\ \kappa_{oc}=\kappa +\frac{2\alpha^{2}}{\omega C} \end{array}\right.$$

the corresponding dispersion relation *D*_oc_ in open-grating is:

$$D_{oc}=\sqrt{\Delta_{oc}^{2}-{\left|\kappa_{oc}\right|}^{2}}$$

Accordingly, the lower and upper edges of the stopband in open-grating for the R-SAW are:

(4) $$\{\begin{array}{c} f_{oc-}=f_{0}\left(1+\frac{2{\left|\alpha \right|}^{2}/\omega C-\left|\kappa_{oc}\right|}{k_{0}}\right)\\ f_{oc+}=f_{0}\left(1+\frac{2{\left|\alpha \right|}^{2}/\omega C+\left|\kappa_{oc}\right|}{k_{0}}\right) \end{array}$$

Thus, the four band-edge frequency Equations (3) and (4) are closely related with the COM parameters (*v*, *κ*, *α* and *C*).

### 2.2. FEM Simulation of Periodic Structures

Usually, the 2-D FEM with COMSOL for SAW devices is implemented on the assumption of plane strain condition, which requires the out-of-plane strain component to be zero. Thus, all the transverse wave solutions are omitted and inaccuracies occur in many piezoelectric structures. Here, we adopted the 3-D FEM model instead. The 3-D FEM models in the study were prepared in COMSOL by two steps. First, a SAW device of the periodic electrodes covering a piezoelectric substrate in the absence of mass loading was modeled. Second, a mass loading layer deposited on the SAW device in the first step was studied for the mass loading effect. The periodic structures were modeled as infinite gratings and only one period was created for simulation purposes. Attention was paid to finding the band-edge frequencies both before and after a mass loading layer deposited on the SAW devices to obtain the COM parameters, respectively.

FEM was used to provide numerical solutions defined by associated differential equations. Propagation of surface acoustic wave in the piezoelectric substrate governed by the coupled wave equations for particle displacements *u_i_* and the potential *Φ* are [15]:

$$\begin{array}{c} \left\{ \begin{array}{l} \rho \frac{\partial^{2}u_{i}}{\partial t^{2}}-c_{ijkl}^{E}\frac{\partial^{2}u_{k}}{\partial x_{l}\partial x_{j}}-e_{kij}\frac{\partial^{2}\Phi }{\partial x_{k}\partial x_{j}}=0\\ e_{jkl}\frac{\partial^{2}u_{k}}{\partial x_{l}\partial x_{j}}-\varepsilon_{jk}^{S}\frac{\partial^{2}\Phi }{\partial x_{k}\partial x_{j}}=0 \end{array}\right.\\ \left(i,j,k,l=1,2,3\right) \end{array}$$

where ρ, $c_{ijkl}^{E}$, $e_{kij}$ and $\varepsilon_{jk}^{S}$ are the density, elastic stiffness tensor, piezoelectric tensor and dielectric permittivity tensor of the substrate, respectively. 

**Figure 3:**
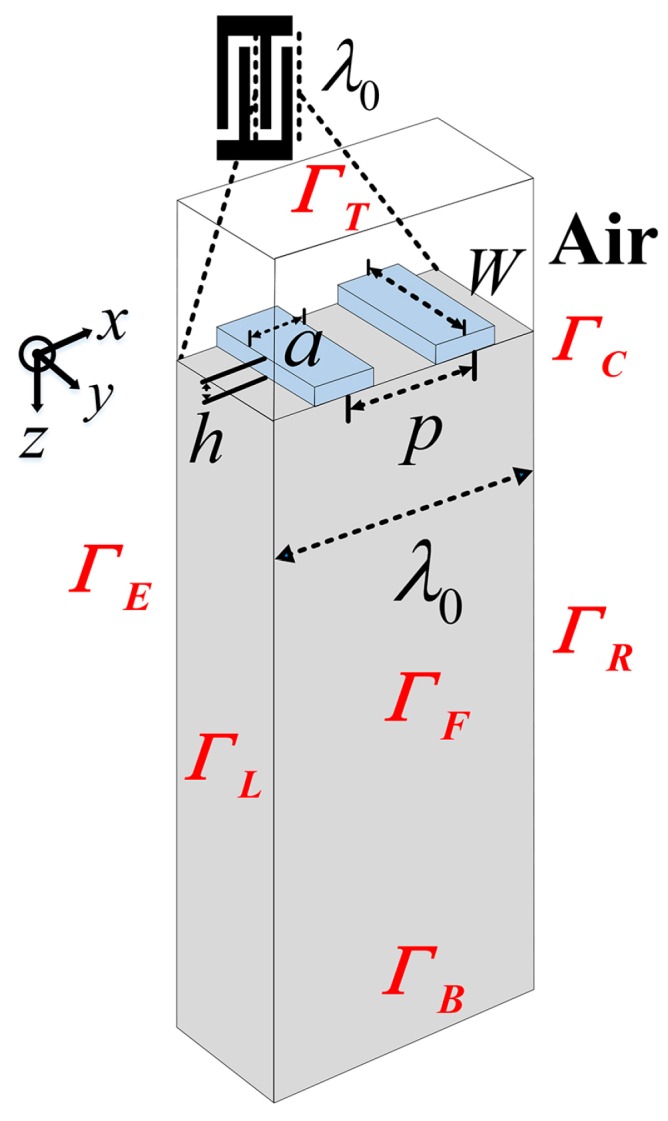
The schematic of the periodic electrodes covering a piezoelectric substrate model.

First, the schematic of the periodic electrodes on a piezoelectric substrate is described in Figure 3. The *x*-direction is taken parallel to the propagation vector and *z* is normal to the interface. There is no variation of the amplitudes in the *y*-direction perpendicular to the sagittal plane (*x*, *z*), which is assumed to be infinitely uniform. The structure mentioned in Figure 3 consists of one pair of aluminum IDT fingers at the surface of a ST-X quartz piezoelectric substrate with the wavelength *λ*_0_ of 10 μm. The depth of the substrate is limited to 4*λ*_0_ and the acoustic aperture *W* is set to 0.5*λ*_0_ to reduce the size of the problem. The metallization ratio (2*a*/*λ*_0_) is 0.5 and the relative thickness of the Al-electrode (*h*/*λ*_0_) is set to 1.6%, where *a* and *h* are the electrode width and height, respectively. As reported by Hofer *et al.*, the charge on the electrode-air interfaces on a quartz substrate (a material with low dielectric constant) has to be taken into account [16], so an air layer with the dielectric constant of 8.854 × 10^−12^ F/m in 0.5*λ*_0_ height was added on the substrate surface of the structure. The material constants of quartz and Al are listed in Table 1 in abbreviated subscript notation [17]. The detailed mechanical and electrical boundary conditions of the modal are listed in Table 2, where the periodic continuity boundary condition stands for all the variables satisfying the Bloch periodic theorem with phase set to zero.

**Figure 4:**
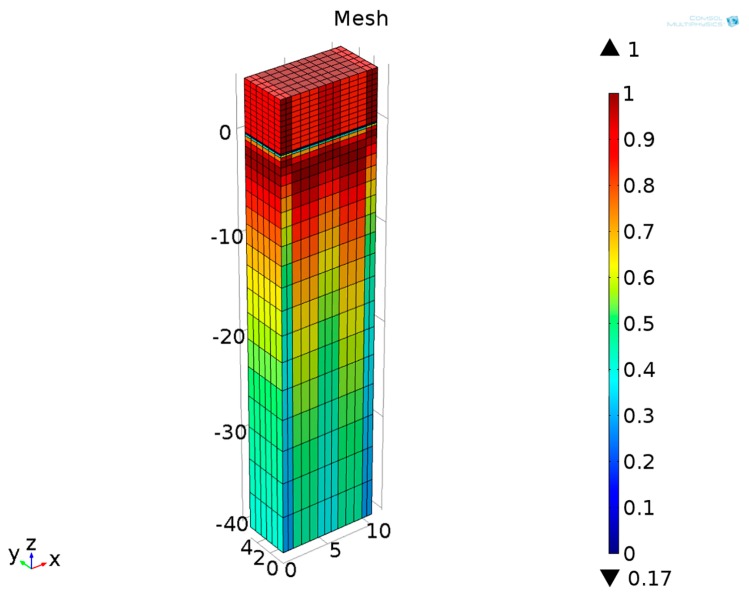
Meshed periodic structure in Figure 3.

The developed model was meshed with the element shape of cube throughout. Since the SAW displacements are largest near the substrate surface, the domain was discretized to higher density near the surface than near the bottom. Besides, the electrodes were meshed to high degree of density. The maximum element size is 1 μm and the complete mesh consists of 2496 domain elements, shown in Figure 4.

**Figure 5:**
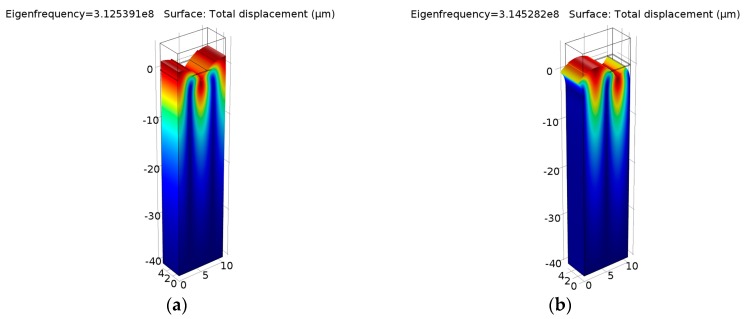
Displacement profiles of periodic shorted-grating on ST-X Quartz: (**a**) eigenfrequency *f*_sc−_ = 312.539 (MHz); (**b**) eigenfrequency *f*_sc+_ = 314.528 (MHz).

The modal analysis was adopted to extract the vibration modes at two eigenfrequencies (*f*_sc−_ and *f*_sc+_), which contribute the edges of the stopband in a periodic shorted-grating [10]. The electrical condition of IDT is set to ground. Actually, the potential is automatically set to zero at the electrodes regardless of the applied values for linear eigenfrequency problems in COMSOL. Figure 5 shows the displacement profiles of the periodic structure. 

At the resonant frequency *f*_sc−_, a zero displacement component in the *x*-direction was observed at both ends. At the anti-resonant frequency *f*_sc+_, the mode has a zero displacement component in the *z*-direction at both ends. Figuring out the two eigenfrequencies, the COM parameters velocity *v* and the amplitude of normalized reflectivity *κλ*_0_ can be extracted by Equation (3), namely:

(5) $$\{\begin{array}{l} v=\lambda_{0}\frac{\left(f_{sc+}+f_{sc-}\right)}{2}\\ \left|\kappa \right|\lambda_{0}=2\pi \frac{f_{sc+}-f_{sc-}}{f_{sc+}+f_{sc-}} \end{array}$$

**Figure 6:**
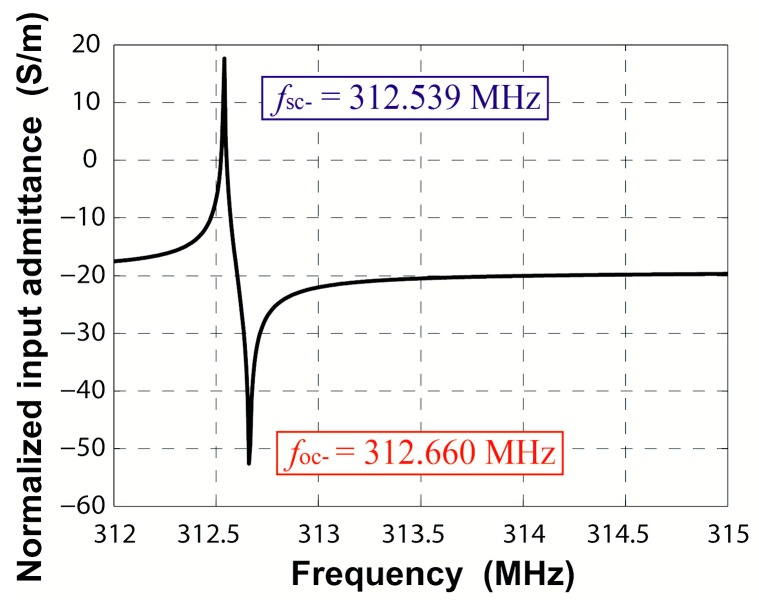
Input admittance of periodic IDT on ST-X quartz.

**Figure 7:**
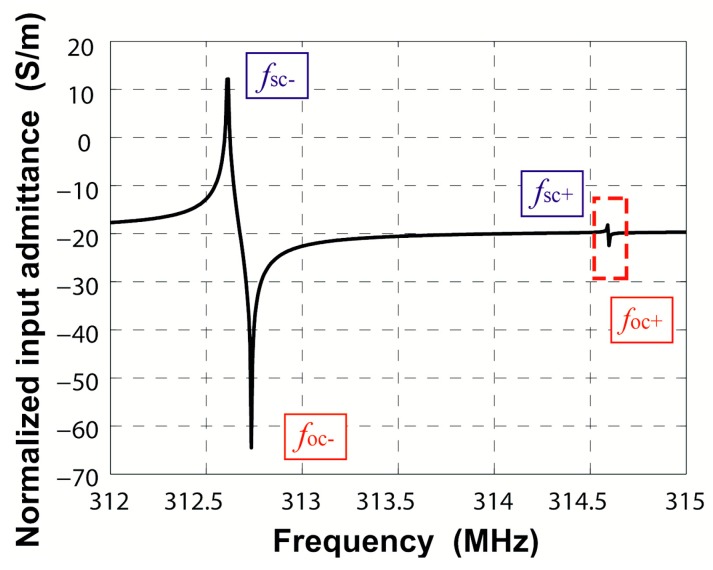
Input admittance of periodic IDT on ST-2°X quartz: the red dashed box area shows the counteracted extrema in Figure 6.

The harmonic frequency response was adopted to extract the harmonic admittance *Y*(*β*, *ω*), where *β* is the wavenumber and *ω* is the operating frequency. The harmonic admittance *Y*(*β*, *ω*) for fixed *β* = 2π/*λ*_0_ corresponds to two times the input admittance *Y*_in_(*ω*) per period for infinite IDT with the period *λ*_0_ [18]. Searching the poles and zeros of the input admittance *Y*_in_ = j*ωQ*/∆*V*, the edges of stopband in periodic shorted-grating and open-grating can be obtained, respectively. Applying alternating voltage drop ∆*V* = 1 V to IDT patterned on the piezoelectric substrate generates the surface acoustic waves. The logarithmic magnitude of the normalized input admittance *Y*_in_/*W* is shown in Figure 6. In this bidirectional structure, only one pair of maximum and minimum values appears, namely another pair of extrema is cancelled out by lack of directivity. The cancelled extrema can be found by adding a tiny directivity to the substrate [18]. Figure 7 gives the input admittance of ST-2°X quartz piezoelectric substrate. Here, the two resonance frequencies present the edges of stopband *f*_sc−_ and *f*_sc+_ in the shorted-grating, which can be demonstrated by the modal analysis described before. The two anti-resonance frequencies present the edges of stopband *f*_oc−_ and *f*_oc+_ in the open-grating. The area enclosed by a red dashed box is the counteracted extrema in Figure 6. The calculated results are consistent with the conclusions in [18].

Based on the Equations (3) and (4), the amplitude of normalized transduction coefficient $\alpha_{n}=\alpha \lambda_{0}/\sqrt{\frac{W}{\lambda_{0}}}$ and the cosine angle between the square of *α* and *κ* can be extracted, namely:

(6) $$\{\begin{array}{l} \left|\alpha_{n}\right|=\sqrt{\omega C_{n}\lambda \pi \left(\frac{f_{oc+}+f_{oc-}}{f_{sc+}+f_{sc-}}-1\right)}\\ \text{cos}\phi =\text{cos}\left(\alpha^{2}/\kappa \right)=\frac{{\left(f_{oc+}-f_{oc-}\right)}^{2}-{\left(f_{sc+}-f_{sc-}\right)}^{2}-{\left[\left(f_{oc+}+f_{oc-}\right)-\left(f_{sc+}+f_{sc-}\right)\right]}^{2}}{2\left(f_{sc+}-f_{sc-}\right)\left[\left(f_{oc+}+f_{oc-}\right)-\left(f_{sc+}+f_{sc-}\right)\right]} \end{array}$$

Besides, the upper edge of the shorted-grating equals the upper edge in the open-grating as shown in Figure 6, namely *f*_sc+_ = *f*_oc+_. Thus, −*κ* = |*κ*|, namely *κ* is negative. From Equation (6), the positive or negative sign of *α* can be determined.

The stationary analysis was adopted to extract the static capacitance. The electrostatic field energy *W*_e_ equals to the energy required for the charge *Q* of a capacitor, namely:

$$W_{e}=\frac{Q^{2}}{2C\lambda_{0}}$$

Besides:

$$C\lambda_{0}=\frac{Q}{\Delta V}$$

where ∆*V* is the voltage drop. Thus, the normalized static capacitance $C_{n}=C\lambda_{0}/W$ is:

(7) $$C_{n}=\frac{2W_{e}}{{\left(\Delta V\right)}^{2}W}$$

In conclusion, all the COM parameters in R-SAW periodic structure shown in Figure 3 were extracted by the FEM.

**Figure 8:**
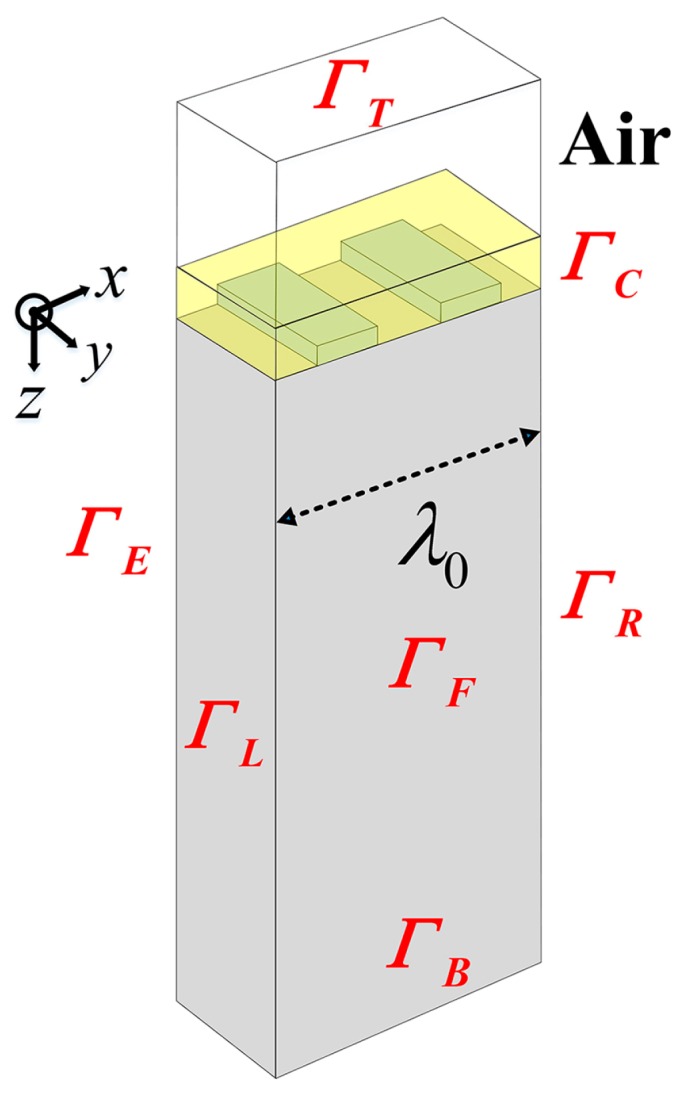
The schematic of a layered periodic model with embedded electrodes.

Additionally, when a mass loading layer is deposited onto the SAW device surface in the first step, the corresponding model of this periodic structure is presented in Figure 8.

**Figure 9:**
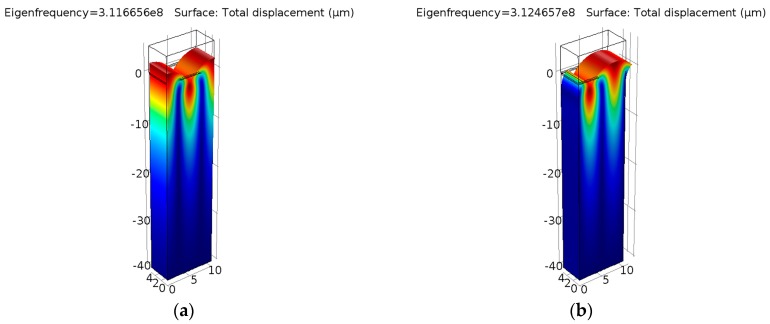
Displacement profiles of periodic layered shorted-grating on ST-X quartz: (**a**) eigenfrequency *f′*_sc−_ = 311.666 (MHz); (**b**) eigenfrequency *f′*_sc+_ = 312.466 (MHz).

**Figure 10:**
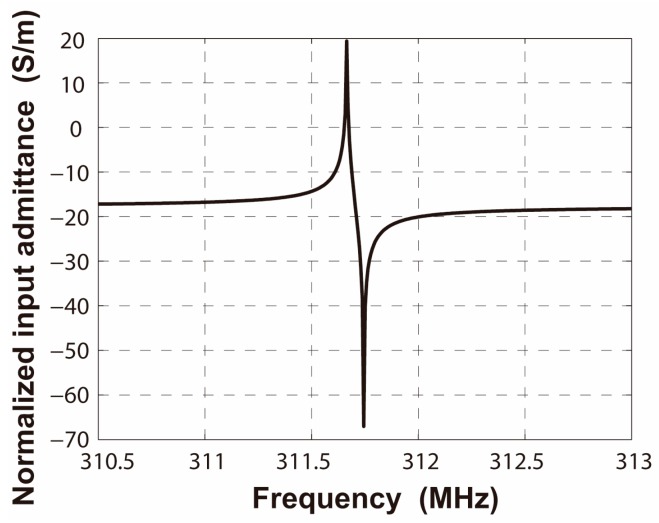
Input admittance of periodic layered IDT on ST-X quartz.

Here, the mass loading layer is assumed to be isotropic SiO_2_ with a height of 0.3 μm, the corresponding material constants are a mass density of 2200 kg/m^3^, Poisson's ratio of 0.17 and the dielectric constant is 36.7 × 10^−12^ F/m [19]. In the same way as the first step, all the COM parameters in a layered R-SAW periodic structure can be obtained easily. The edges of stopband *f′*_sc−_ and *f′*_sc+_ in a layered shorted-grating can be derived from the displacement profiles by the modal analysis, as shown in Figure 9. The edges of stopband *f′*_oc−_ and *f′*_oc+_ in a layered open-grating can be extracted from the normalized input admittance by the harmonic frequency response, which is shown in Figure 10. Finally, using the stationary analysis, the normalized static capacitance can be obtained.

Thus, the layered periodic structure shown in Figure 8 can be simulated easily by using COMSOL. All the COM parameters in the mass loaded periodic structure were extracted in the same way as the first step without complicated operations. The extracted COM parameters are listed in Table 3. Based on the COM parameters in the absence and presence of a mass loading layer covering the electrode structures, the frequency responses of the R-SAW resonators without and with mass loading are studied in the following section.

### 2.3. Frequency Responses of R-SAW Resonators

**Figure 11:**
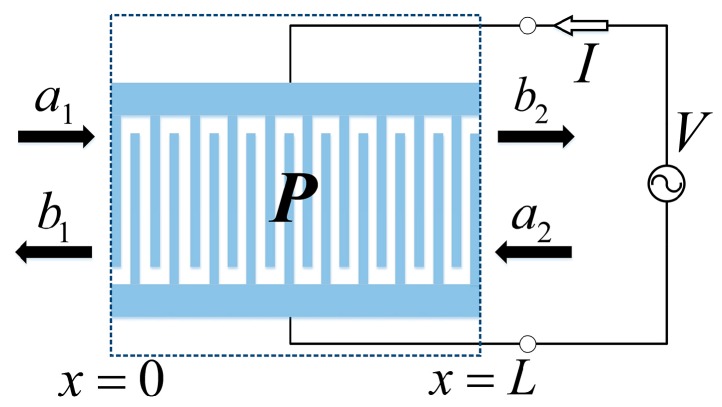
The schematic of the *P*-matrix in the IDT section.

To cascade the uniform transducer elements, solutions of the COM equations can be derived in the *P*-matrix [13]. Figure 11 shows the schematic diagram of the *P*-matrix in the IDT element. In the *P*-matrix, the two acoustic ports’ relationship is described by the scattering matrix and the electrical port relationship is described by the admittance matrix as follows:

(8) $$\left(\begin{array}{c} b_{1}\\ b_{2}\\ I \end{array}\right)=\left(\begin{array}{ccc} P_{11} & P_{12} & P_{13}\\ P_{21} & P_{22} & P_{23}\\ P_{31} & P_{32} & P_{33} \end{array}\right)\left(\begin{array}{c} a_{1}\\ a_{2}\\ V \end{array}\right)$$

where:

$$\left\{ \begin{array}{c} P_{11}=\frac{j\kappa^{*}\text{sin}\left(DL\right)}{D\text{cos}\left(DL\right)+j\Delta \text{sin}\left(DL\right)}\\ P_{12}=P_{21}=\frac{{\left(-1\right)}^{2N}D}{D\text{cos}\left(DL\right)+j\Delta \text{sin}\left(DL\right)}\\ P_{13}=-\frac{1}{2}P_{31}=jL\frac{\text{sin}\left(DL/2\right)}{DL/2}\frac{j\left(\Delta \alpha^{*}+\kappa^{*}\alpha \right)\text{sin}\left(DL/2\right)+\alpha^{*}D\text{cos}\left(DL/2\right)}{D\text{cos}\left(DL\right)+j\Delta \text{sin}\left(DL\right)}\\ P_{22}=\frac{j\kappa \text{sin}\left(DL\right)}{D\text{cos}\left(DL\right)+j\Delta \text{sin}\left(DL\right)}\\ P_{23}=-\frac{1}{2}P_{32}={\left(-1\right)}^{2N}jL\frac{\text{sin}\left(DL/2\right)}{DL/2}\frac{j\left(\Delta \alpha +\kappa \alpha^{*}\right)\text{sin}\left(DL/2\right)+\alpha D\text{cos}\left(DL/2\right)}{D\text{cos}\left(DL\right)+j\Delta \text{sin}\left(DL\right)}\\ \begin{array}{cc} P_{33}= & -\frac{4}{D^{3}}\frac{\left[\left(\Delta^{2}+{\left|\kappa \right|}^{2}\right){\left|\alpha \right|}^{2}+2\Delta \text{Re}\left(\kappa^{*}\alpha^{2}\right)\right]\left[1-\text{cos}\left(DL\right)\right]}{D\text{cos}\left(DL\right)+j\Delta \text{sin}\left(DL\right)}\\ & +j\frac{4}{D^{2}}\frac{\left[\Delta {\left|\alpha \right|}^{2}+\text{Re}\left(\kappa^{*}\alpha^{2}\right)\right]\text{sin}\left(DL\right)}{D\text{cos}\left(DL\right)+j\Delta \text{sin}\left(DL\right)}-jL\frac{4}{\Delta^{2}-{\left|\kappa \right|}^{2}}\left[\Delta {\left|\alpha \right|}^{2}+\text{Re}\left(\kappa^{*}\alpha^{2}\right)\right]+j\omega LC \end{array} \end{array}\right.$$

*L* is the device length and *N* is the pair number of electrodes, namely *L* = *Nλ*_0_. Re is the real part of the extraction operator. Substituting the COM parameters (*v*, *κ*, *α* and *C*) extracted by FEM into Equation (8), the values of *P*-matrix elements are given. More detailed derivation of the *P*-matrix was presented in reference [13].

**Figure 12:**
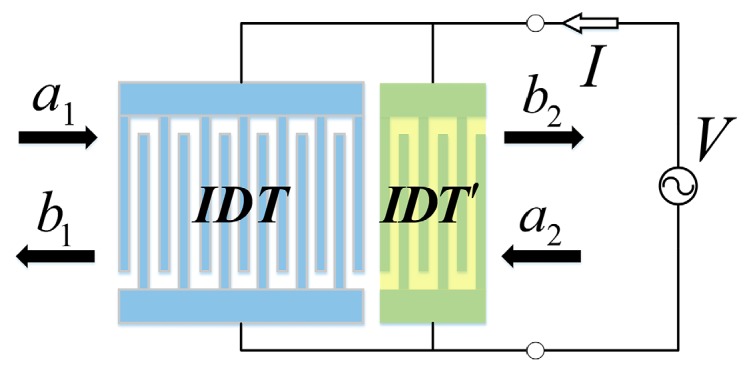
The schematic of *P*-matrix in IDT section.

Omitting the source item in Equation (8), that is only the scattering matrix of the two acoustic ports relationship is considered, the *P*-matrixes of the gratings and gaps are obtained. When the mass loaded IDT′ is considered as shown in Figure 12, the *P*-matrix in IDT′ is determined by using the COM parameters from the layered structure in step 2. The cascading relationships of different elements are the acoustic ports cascaded and the electrical ports in parallel, which complete the analysis of the SAW resonator with mass loaded on different sensitive areas.

**Figure 13:**
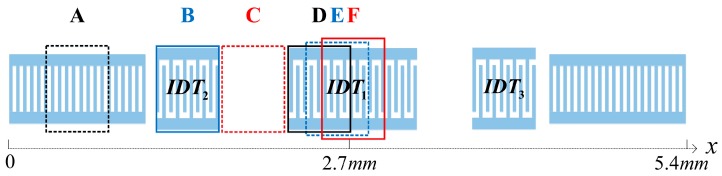
The schematic of mass deposited along the *x*-axis from A to F on the two-port SAW resonator.

**Figure 14:**
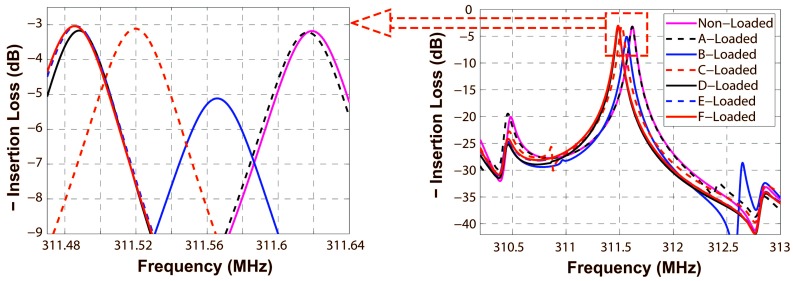
The frequency responses for non-loaded and different sensitive areas shown in Figure 13 with mass loaded of the resonator.

Utilizing the *P*-matrix model, the frequency response S_21_ of a two-port R-SAW resonator structure was calculated. When the mass covers different sensitive areas from A to F, shown in Figure 13, the corresponding center frequency perturbations are determined by finding the maximum amplitude of S_21_. As the resonator structure is symmetrical, only half of the surface was chosen for the analysis. The rectangular frames of different size are just schematic. Deposited areas are all the same in practice, namely 20*λ*_0_ in the *x*-direction, 150*λ*_0_ in the *y*-direction and 3000 Å high. Figure 14 shows the frequency responses S_21_ around the center frequency (311.6 MHz) for non-loaded device and different sensitive areas with mass loaded shown in various colour and line styles. The electrode number of IDT_1_, IDT_2_ and IDT_3_ are 90, 45 and 45, respectively. The center frequencies are reduced for the mass loading effect.

Using the mass sensitivity *S*_m_ equation defined by [9] as:

(9) $$S_{m}=\frac{f_{m}-f_{0}}{m}$$

where *f*_m_ is the perturbed center frequency, *f*_0_ is the unperturbed center frequency and *m* is the total mass of the perturbing material. The method proposed in the paper can be implemented to calculate the mass sensitivity changes in different sensitive areas of an R-SAW resonator for sensor applications.

## 3. Technique Realization

### 3.1. R-SAW Resonator Preparation

**Figure 15:**
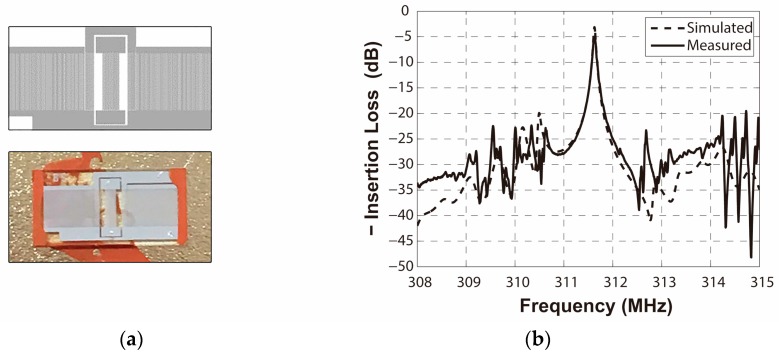
(**a**) The structure of the SAW resonator device; (**b**) the frequency response of the device.

The two-port R-SAW resonator was fabricated on the ST-X quartz substrate by a standard photolithographic technique, on which 1600 Å aluminum IDTs and adjacent shorted grating reflectors were deposited. The operation frequency of the resonator was designed as 311.6 MHz, thus the corresponding wavelength *λ*_0_ was 10 μm. The number of electrodes of the launching transducers IDT_2_, IDT_3_, and the reading transducer IDT_1_ are 45, 45 and 90, respectively. The number of electrodes of the reflectors are both set to 400. The acoustic aperture is 150*λ*_0_. The cavity between the IDT and adjacent reflectors is 1.25*λ*_0_, and the cavity between the IDTs is 20.25*λ*_0_, which provide lower insertion loss and high Q-value. The frequency response S_21_ of the two-port resonator was measured by using an Agilent E5071B Network Analyzer. Figure 15 shows the fabricated SAW device and the corresponding frequency response S_21_. A low insertion loss of 4 dB and center frequency of 311.625 MHz device was obtained in accordance with the simulated result (insertion loss of 3 dB and center frequency of 311.625 MHz).

### 3.2. Regional Mass Loading

SiO_2_ was chosen as the added mass material, whose influence on the SAW device is mainly a mass loading effect rather than any viscoelasticity effect. Combining the photolithographic technique to determine the deposited position and RF magnetron sputtering to deposit SiO_2_, the different mass loaded areas across the surface were guaranteed by a lift-off procedure. The deposited SiO_2_ area is 20*λ*_0_ in the *x*-direction, 150*λ*_0_ in the *y*-direction and 3000 Å high, which was measured by an Alpha-Step IQ Surface Profiler. Each deposited position was repetitively fabricated on twenty different devices in the same layout. 

**Figure 16:**
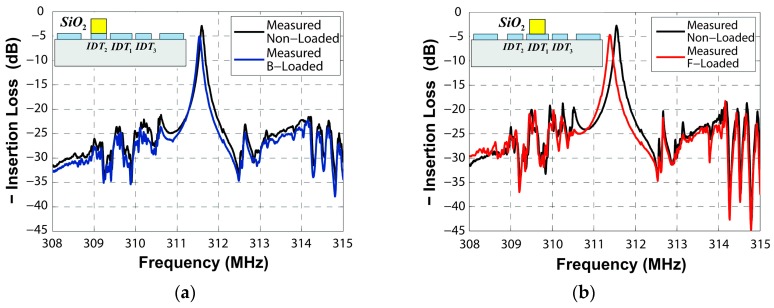
Measured frequency responses caused by (**a**) area B loaded and (**b**) area F loaded by SiO_2_.

Figure 16a,b exhibit the influence of SiO_2_ deposited on areas B and F on similar devices, measured by the network analyzer. The frequency changes caused by mass loading areas B and F are 62 KHz and 166 KHz, respectively.

## 4. Results and Discussion

**Figure 17:**
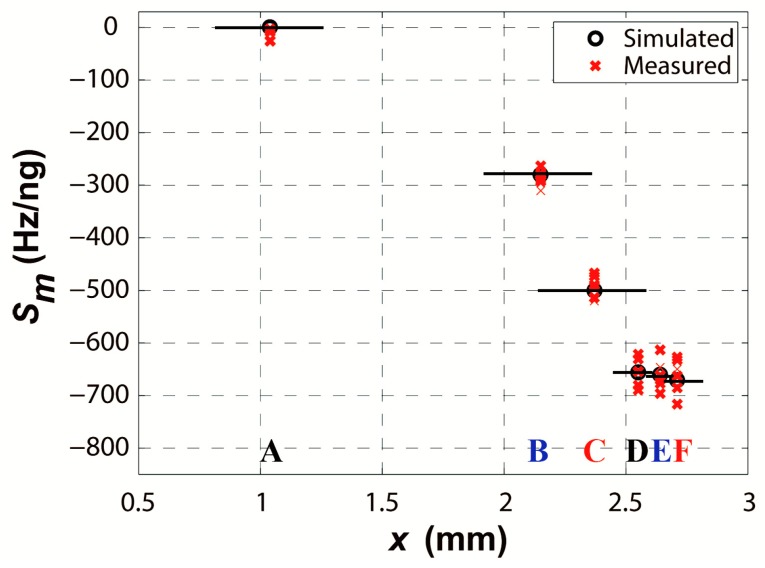
The simulated and measured mass sensitivity for different surface areas, each position being demonstrated by repeated measurements.

Then, all the measurements were implemented in the air environment by using the network analyzer and RF probes. Figure 17 gives the measured mass sensitivity for different surface areas of the R-SAW resonator. The horizontal axis presents the length of the device covering the area from A to F, corresponding to the schematic of Figure 13. The values on the vertical axis are negative as the frequencies are reduced by the mass loading effect. The circles are the simulated results, which are decorated with short solid lines for clearness. The crosses are the measured results.

It is shown that the optimal sensitive area is focused on the center IDT, which just occupies 8% of the whole surface. The sensitivity variation for different areas D–F in the center IDT seems slight, but their sensitivities are more than two times that in the lateral IDT (B). The reflector (A) contributes little to the mass sensitivity. Thus, we need pay great attention to the optimal sensitive area instead of the whole surface in practice. The results show a similar variation as described by Powell, due to the energy concentration towards the center area by the resonators.

In the paper, FEM has provided an easy way to extract COM parameters. A computer with an i5 CPU and 16 GB memory was used to simulate FEM models with COMSOL. The whole COM parameter extraction process in a fixed structure will take two hours, which is acceptable for practical applications. However, the extraction using FEM has limitations. It doesn’t work if the SAW under consideration is very lossy or dispersive, such as leaky wave and STW wave devices. The resonances of the periodic structures won’t be given simply as Equations (3) and (4).

## 5. Conclusions

This paper provides the theory and experimental analysis of the mass sensitivity variation for different sensitive areas of an R-SAW resonator. By using the FEM with COMSOL, an easy and fast way for COM parameter extraction was proposed, even for layered structures. The theoretical results were obtained to determine the optimal sensitivity area and verified by the measured responses. The optimal sensitivity area of a two-port R-SAW resonator with three IDTs is the center IDT. The sensitivity variation is strong across the surface. The results in this paper can guide the detection position choice for SAW sensors.

## Figures and Tables

**Table 1:** Material constants for quartz and Al.

Material	Density (kg/m^3^)	Elastic Stiffness Constant (10^10^ N/m^2^)	Piezoelectric Stress Constant (C/m^2^)	Dielectric Constant (10^−12^ F/m)
Quartz	2651	*c*_11_ = 8.674	*e*_11_ = 0.171	*ε*_11_ = 39.843
		*c*_12_ = 0.699	*e*_14_ = −0.0436	*ε*_33_ = 40.7284
		*c*_13_ = 1.191		
		*c*_14_ = −1.791		
		*c*_33_ = 10.72		
		*c*_44_ = 5.794		
		*c*_66_ = 3.9875		
Al	2700	*c*_12_ = 5.11		
		*c*_44_ = 2.63		

**Table 2:** Boundary conditions of the model.

Boundary	Mechanical Boundary Conditions	Electrical Boundary Conditions
*Γ*_T_ (top of air)		Zero charge
*Γ*_C_ (air-solid interfaces)	Free	Continuity
*Γ*_B_ (bottom of substrate)	Fixed	Ground
*Γ*_L_, *Γ*_R_ (left and right boundaries)	Periodic continuity boundary condition	
*Γ*_F_, *Γ*_E_ (front and back boundaries)	Periodic continuity boundary condition	

**Table 3:** COM parameters extracted by FEM for the structures without and with mass loading.

COM-Parameter	Value (without Mass Loading)	Value (with Mass Loading)
SAW Velocity (m/s)	3149.1	3134.4
Normalized reflectivity *κλ*_0_	−0.020	−0.008
Normalized transduction coefficient *α* (Ω^−1/2^)	2.573 × 10^−5^	2.186 × 10^−5^
Normalized static capacitance *C*_n_ (F/m)	5.52 × 10^−11^	5.74 × 10^−11^
